# Peroneal Artery–based Propeller Flap to Cover the Medial Distal Tibia in the Absence of the Posterior Tibial Artery

**DOI:** 10.1097/GOX.0000000000002354

**Published:** 2019-10-30

**Authors:** Alexander J. Kish, Raymond A. Pensy

**Affiliations:** From the Department of Orthopaedics, R Adams Cowley Shock Trauma Center, Baltimore, Md.

## Abstract

A healthy 28-year-old woman restrained driver presented to the trauma unit post-MVC with significant vehicular intrusion. Examination demonstrated a 15-cm transverse wound over the medial malleolus and anterior ankle with exposed muscle, tendon, and bone without gross contamination. Her physical examination was otherwise unremarkable. Distal to the wound, there was no Doppler signal in either dorsalis pedis or posterior tibial arteries and the foot appeared cold with delayed cap refill. She was taken to the operating room urgently for debridement and irrigation, open reduction internal fixation of both distal tibia and fibular fractures, and supplemental external fixation application. The foot regained a normal color and capillary refill upon reduction, and biphasic Doppler signals returned.

Open lower extremity fractures with associated arterial injury are devastating. Even in the absence of absolute ischemia, limb salvage is difficult to achieve, as disruption of one more axial vessels severely complicates the soft tissue reconstructive options. These injuries, frequently associated with ischemic damage and reperfusion injury, are associated with flap failures and subsequently carry up to a 75% risk of amputation.^1,2^ Despite this, significant advances in limb salvage have been made, particularly in regard to soft tissue coverage options.

In 1989, the first description of a perforator-based flap was outlined by Koshima and Soeda^3^ using a musculocutaneous flap with inferior epigastric artery-based skin islands to reconstruct soft tissues of the face and mouth. This description led to many advancements in the understanding of vascular anatomy and laid the framework for the development of propeller flaps. Initially described by Hyakusoku et al,^4^ the propeller flap is a local island fasciocutaneous flap based on a single dissected perforator. Two limbs are created of unequal size with the perforator vessel creating the pivot point so the larger limb of the flap can be rotated up to 180 degrees to fill a larger deficit. This flap design represents an increasingly popular option for soft tissue coverage of the tibia.

The distal lower extremity provides a challenge for soft tissue reconstruction after loss of substance, subsequent to the sparsity of surrounding soft tissue amenable for transfer. Traditionally, proximally based peninsula fasciocutaneous flaps in the lower extremity have proven to be generally ineffective, secondary to the inability to provide adequate wound coverage and resultant exposure of critical areas like the subcutaneous border of the tibia or Achilles tendon.^5^ For this reason, propeller flaps have become increasingly popular because they provide local coverage to soft tissue defects about the distal lower extremity. Propeller flaps simply require an axial vessel with an available and suitable perforator and careful dissection. Importantly, defect closure is achieved without the need for microvascular anastomosis, sacrifice of the axial vessel, or its overall arterial supply to the distal extremity.

The vascular anatomy of the lower extremity is complex, and multiple variations have been reported. Thus, a familiarity of these variations is imperative when planning advanced soft tissue reconstructions. In the majority of specimens, the popliteal artery bifurcates into the anterior tibial and tibioperoneal trunk, the latter then bifurcating into the peroneal and posterior tibial vessels. In up to 5% of cases, the peroneal artery replaces the posterior tibial vessels.^6^ As the posterior tibial artery is the most common recipient vessels for lower extremity free tissue transfer and the basis for medially based propeller flaps, its absence in conjunction with traumatic defects of the medial leg presents can be an expected challenge worthy of consideration.^7^

## CASE REPORT

A healthy 28-year-old woman restrained driver presented to the trauma unit post-MVC with significant vehicular intrusion. Examination demonstrated a 15-cm transverse wound over the medial malleolus and anterior ankle with exposed muscle, tendon, and bone without gross contamination. Her physical examination was otherwise unremarkable. Distal to the wound, there was no Doppler signal in either dorsalis pedis or posterior tibial arteries and the foot appeared cold with delayed cap refill. She was taken to the operating room urgently for debridement and irrigation, open reduction internal fixation of both distal tibia and fibular fractures, and supplemental external fixation application. The foot regained a normal color and capillary refill upon reduction, and biphasic Doppler signals returned.

**Fig. 1.**
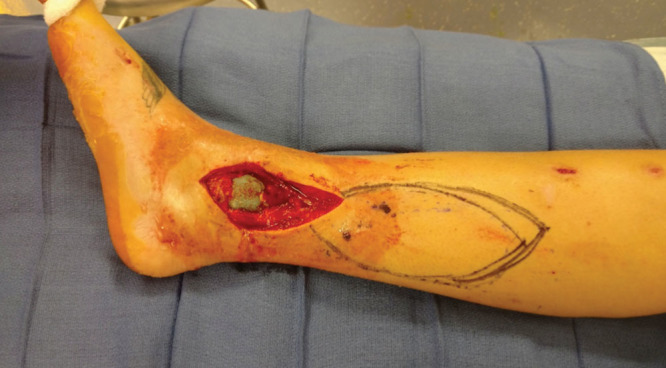
Post irrigation and debridement with antibiotic spacer placement, there was approximately a 10 × 14 cm soft tissue defect over the medial ankle.

The patient did well postoperatively and was discharged home on postoperative day 6. At her 6-week postoperative visit, the external fixation was removed and she was placed in a well-padded short leg cast. At postoperative week 9, the patient returned to clinic after having fevers and when the cast was removed and a 1.5 × 2.5 cm area of superficial central wound dehiscence was observed without surrounding erythema or fluctuance. She was prescribed Bactrim and placed in another short leg cast. At postoperative week 10, the cast was removed demonstrating exposed bone and hardware in the central aspect of the wound and the patient was admitted to the hospital for debridement and removal of implants with antibiotic spacer placement. At this time, there was a 10 × 14 cm wound over the medial malleolus (Fig. 1).

**Fig. 2.**
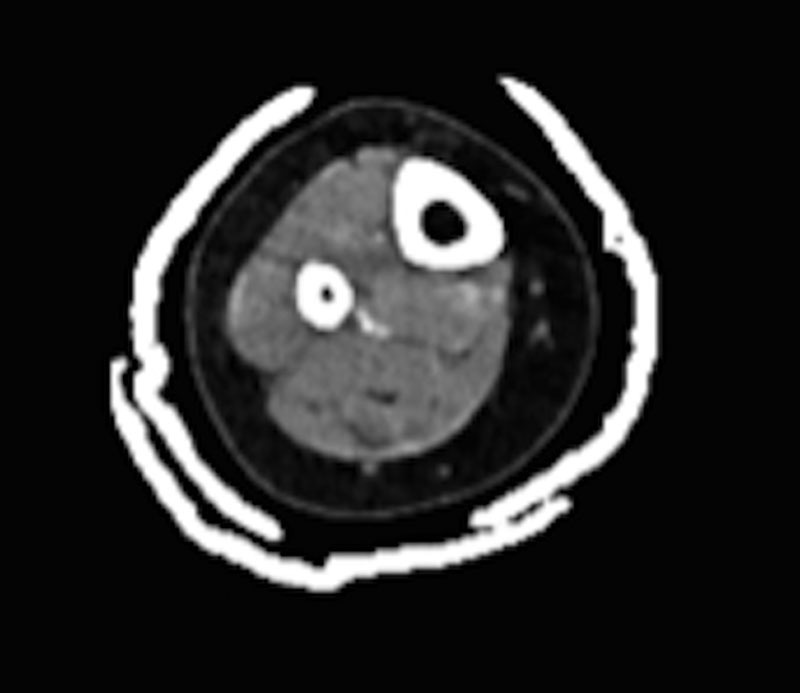
CTA demonstrating laterally based peroneal artery perforator supplying medial sided skin. Perforator coursing medially between deep and superficial posterior compartments.

As part of routine flap planning for open fractures, a formal angiogram was obtained. This demonstrated congenital absence of the posterior tibial artery. A large peroneal artery and a normal anterior tibial artery were noted. This anatomic variation is found in 5% of the normal population.^6^ A perforator supplying the medial skin, well proximal to the zone of injury, was identified via the CTA, completed upon presentation weeks earlier (Fig. 2). The perforator to the medial skin, proximal to the defect and arising from the peroneal artery, was again visualized emanating from the peroneal artery, coursing between the deep and superficial compartments to the medial skin.

In this case, free tissue was deemed a relative contraindication because the patient demonstrated a single vessel supplying the foot. A propeller flap, based on the perforator from the peroneal artery, was chosen to provide medial soft tissue coverage. As noted, preoperative CTA and angiography demonstrated a suitable peroneal perforator providing flow to medial skin, in the absence of the PT artery.

Intraoperatively, after the perforator was localized with pencil Doppler, laser-assisted endocyanin green angiography was used to confirm the location of perforator.

**Fig. 3.**
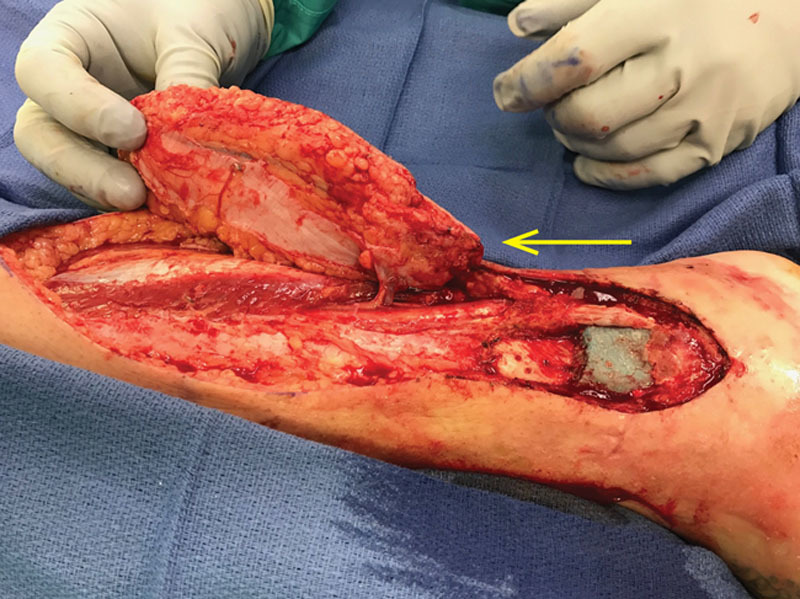
Flap elevated with perforator highlighted by yellow arrow.

**Fig. 4.**
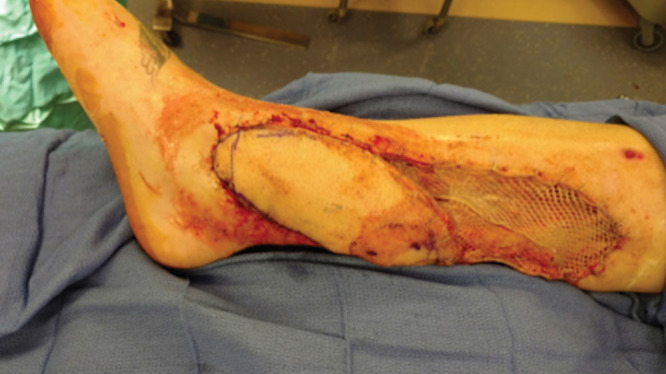
Propeller flap in final position. Proximal defect covered with split thickness skin grafting.

An anterior exploratory incision was made, just posterior to the tibial border. The fascia was elevated off deep posterior compartment, and upon reaching septa between the deep and superficial compartments of the leg, the perforator was easily visualized superficial to the flexor digitorum longus muscle. The perforator was found and dissected in the typical plane of the medial perforators, that being between the superficial and deep compartments. As expected from preoperative imaging, this perforator had its origin from the peroneal artery, found lateral to the tibial nerve, as opposed to the posterior tibial artery, typically found medial to the nerve. A lengthy perforator was isolated as the pedicle to the skin island, totaling slightly more than 4 cm in length, substantially longer than most perforators in those location. After the pedicle was isolated, the flap was rotated on its long axis approximately 160 degrees. The proximal skin defect was covered with split thickness skin graft harvested from the ipsilateral thigh (Figs. 3, 4). The patient underwent routine flap care with strict elevation and a Bair hugger over the affected extremity. The patient was discharged to home on postoperative day 4.

## DISCUSSION

The posterior tibial artery is absent in 1–5% of the normal population.^6^ Compensatory hypertrophy of the peroneal artery can be associated with hypoplastic or aplastic posterior tibial artery and has been described as the “perona magna.” When this is observed, the distal aspect of the peroneal artery continues on as the lateral plantar artery and the medial plantar artery is absent.^8^ In this case, a reconstructive challenge presented in a patient with loss of the distal medial tibia soft tissue coverage subsequent to an acute open fracture and a severely injured medial soft tissue envelope, in the setting of a congenitally absent PT.

Free transfer tissue for medial soft tissue coverage of the leg could have been completed using either the peroneal artery as the recipient vessel, an end-to-side anastomosis to the remaining anterior tibial vessel, or a flow through flap (eg, anterolateral thigh) in the anterior system. However, these would have added significant technical difficulty to the procedure, considering potential size mismatch and the design of the skin paddle. Alternatively, a medially based propeller flap, based on the peroneal artery, was chosen. To the author's knowledge, this is the first example of medial propeller flap based on the peroneal artery.

The perforasome theory was initially described by Saint-Cyr et al,^9^ who demonstrated the ability of a single arterial perforator to adequately vascularize large volumes of skin for soft tissue reconstruction. Since the implementation of the propeller flap, it has become increasingly popular as a soft tissue coverage option, particularly for the medial aspect of the lower third of the leg, where free tissue has traditionally been the mainstay for substantial defects.

The propeller flap is not without complications. A recent meta-analysis of pedicled flaps demonstrated a partial skin necrosis rate of 10%, total flap necrosis of 3%, but overall coverage success similar to failure rates of free tissue transfer.^10^ Although partial necrosis was found to be higher in the propeller flap group, the overall success of coverage was found to be not statistically different. In light of the existing literature and the author's experience, careful consideration must be given when choosing between propeller and free tissue transfer. Small defects, supple and nontraumatized skin, a perforator localized to an area adjacent but not within the zone of injury (as evidence by induration, edema, erythema), youthful age, large perforator caliber, all favor the success of the propeller flap.

In the absence of the posterior tibial vessel, it is the author's experience that the medial skin of the lower extremity is supplied via perforators taking origin from the peroneal vessel, and coursing in the typical septal path, between the deep and posterior compartments. In this case, with the available preoperative angiography and CTA, we were able to confirm the precise location of this sizable perforator in area suitable for transfer and provide a readily available, and comparably easy, method for achieving soft tissue coverage for a difficult area of the leg in an otherwise challenging reconstructive dilemma.

This case serves as an illustration that in the congenital absence of the posterior tibial artery, propeller flaps may still play a role in providing medial soft tissue coverage and should be considered as a viable option with appropriate preoperative evaluation.
